# Ultraviolet Spectral Irradiance Scale Comparison: 210 nm to 300 nm

**DOI:** 10.6028/jres.103.001

**Published:** 1998-02-01

**Authors:** Ambler Thompson, Edward A. Early, Thomas R. O’Brian

**Affiliations:** National Institute of Standards and Technology, Gaithersburg, MD 20899-0001

**Keywords:** irradiance, spectral irradiance, synchrotron radiation, ultraviolet radiometry, ultraviolet standards

## Abstract

Comparison of the irradiances from a number of ultraviolet spectral irradiance standards, based on different physical principles, showed agreement to within their combined standard uncertainties as assigned to them by NIST. The wavelength region of the spectral irradiance comparison was from 210 nm to 300 nm. The spectral irradiance sources were: an electron storage ring, 1000 W quartz-halogen lamps, deuterium arc lamps, and a windowless argon miniarc.

## 1. Introduction

Many national programs and industrial applications require accurate measurement of radiation in the ultraviolet (UV) spectral region. Examples of the important applications of UV radiation and measurement technology are: monitoring atmospheric ozone, UV curing of polymers, water purification, and photolithography of semiconductor materials. Artifacts or facilities for transferring absolute UV irradiance are available from the National Institute of Standards and Technology, and are derived from different physical principles. This UV measurement capability is maintained by three organizational divisions within the Physics Laboratory at NIST: the Optical Technology Division (OTD), the Atomic Physics Division (APD), and the Electron and Optical Physics Division (EOPD). The absolute detector-based scales are traceable to fundamental electrical units using the OTDs High Accuracy Cryogenic Radiometer [1]. The source-based scales are derived from blackbody radiation in the OTDs Facility for Automated Spectroradiometric Calibration (FASCAL) [2,3], from plasma sources (i.e., wall-stabilized hydrogen and blackbody arcs) in the APD [4], and from electron storage ring synchrotron radiation in the EOPDs Synchrotron Ultraviolet Radiation Facility II (SURF II) where spectral irradiance and radiance are calculable [5] from the approximations of Schwinger [6]. SURF II is currently undergoing a major upgrade which will lower its overall uncertainties and incorporate the cryogenic radiometer capabilities from the OTD [7].

An ultraviolet intercomparison was recently completed by the Comité Consultatif de Photometrie et Radiometrie (CCPR) of the Comitté International des Poids et Mesures (CIPM) between NIST, the National Physical Laboratory (NPL) of the United Kingdom, and the Physikalische Technische Bundesanstalt (PTB) of Germany [8]. The intercomparison covered spectral radiance and irradiance across the wavelength range 200 nm to 400 nm using deuterium and tungsten-halogen lamps as transfer standards. This intercomparison produced anomalous results in which UV spectral irradiance measurements performed by the different laboratories disagreed with each other by 5 % to 10 %. The NIST scales of UV spectral irradiance have been compared previously: SURF II irradiance was compared with tungsten-halogen standards [9] and with an argon miniarc [10]. The spectral irradiance comparison of SURF II with tungsten-halogen lamps was done at two wavelengths (254 nm and 297 nm) and agreement between the spectral irradiances was on the order of 1 %, well within the relative combined standard uncertainties. Comparison of SURF II to the argon miniarc at 214 nm found agreement to within 3 %, also within the relative combined standard uncertainty of the comparison. Since the results from the latest CCPR intercomparison were at odds with previous measurements at NIST, it seemed worth investigating whether the NIST values of spectral irradiance from sources derived from different physical principles were still in agreement. Therefore the spectral irradiance sources at NIST were compared to determine if unknown systematic effects in the derivation or transfer of the NIST spectral irradiance scales could contribute to the observed differences in the CCPR intercomparison.

## 2. Approach

The primary experimental considerations in comparing the spectral irradiance values from NIST sources were to: 1) carry out the irradiance comparison in air, 2) design the transfer spectroradiometer to minimize known differential source properties, 3) assume short term stability of the spectroradiometer used to compare source spectral irradiances, and 4) design the experiment so as to minimize alignment errors and the need for instrument repositioning. In this regard, the NIST spectral irradiance sources are used to determine the spectral responsivity of a spectroradiometer, in precisely the same manner as any user of standard sources. The spectral responsivity of the instrument should be invariant with the irradiance source, if the design of the spectroradiometer has made it relatively insensitive to geometric and radiometric differences in the standard sources.

The basis for performing this type of evaluation is the simplified measurement equation [11],

$$S(\lambda_{0})=\int E(\lambda )R(\lambda_{0},\lambda )d\lambda ,$$ (2.1)

where λ is a wavelength in the range of a monochromator set at λ_0_, *S* (λ_0_) is the output signal of the photon counting circuit, *E*(λ) is the spectral irradiance of the source, and *R* (λ_0_, λ) is the spectral irradiance responsivity function of the instrument. Further, as shown in Ref. [11] the spectral irradiance responsivity function is given by

$$R(\lambda_{0},\lambda )=R(\lambda )z(\lambda -\lambda_{0}),$$ (2.2)

where *R*(λ) is the irradiance response function and *z*(λ − λ_0_) is the dimensionless slit-scattering function. Combining Eqs. (2.1) and (2.2) yields

$$S(\lambda_{0})=\int E(\lambda )R(\lambda )z(\lambda -\lambda_{0})d\lambda ,$$ (2.3)

The spectral irradiance response function usually varies slowly with wavelength, and indicates the sensitivity of the instrument to light at a given wavelength. Conversely, the slit-scattering function is a rapidly varying function of wavelength. Ideally it is independent of λ_0_ and triangular in shape, and indicates the bandwidth Δλ of the instrument. Given a source with a known spectral irradiance *E_s_* (λ), and assuming that *E_s_* (λ) and *R*(λ) vary slowly over the wavelength range for which *z*(λ − λ_0_) is appreciable, Eq. (2.3) becomes

$$S(\lambda_{0})=E_{s}(\lambda_{0})R(\lambda_{0})\int z(\lambda -\lambda_{0})d\lambda .$$ (2.4)

The product *R* (λ_0_) ∫ *z* (λ − λ_0_)dλ is the spectral irradiance responsivity, and reduces to *R* (λ_0_)dλ for a double monochromator viewing standard sources, whose spectral irradiance changes slowly relative to the instruments bandpass. NIST sources with known spectral irradiances were used to determine the spectral irradiance responsivity of the spectroradiometer in this experiment and the resultant spectral responsivities were compared.

A number of NIST UV irradiance sources with different physical bases for the assignment of spectral irradiance were used in this comparison. The SURF II spectrometer calibration beamline, designated number 2 was used as a spectral irradiance source based on synchrotron radiation and is hereafter referred to as SURF. Two types of spectral irradiance standard lamps based on blackbody radiation and calibrated in FASCAL were used and are designated FEL and D_2_. The first type is a modified 1000 W quartz-halogen FEL type lamp with coiled-coil filaments calibrated from 250 nm to 2400 nm, while the second is a deuterium lamp calibrated from 200 nm to 350 nm. Two FEL lamps and three D_2_ lamps were used in the comparison. The miniature argon arc source also known as an argon miniarc, designated ArArc, was calibrated at NIST by the APD just prior to the comparison. The comparison of the ArArc, FEL and D_2_ with SURF was carried out in air since the ArArc, FEL, and D_2_ standards are calibrated in air and many UV applications require in-air calibrations.

**Fig. 1:**
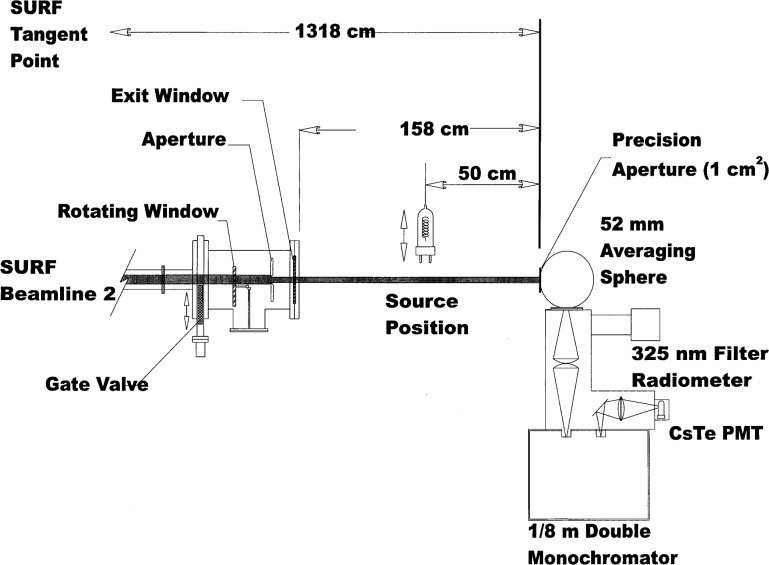
Schematic diagram of the experimental setup on beamline 2 of the NIST SURF II. The diagram shows the relative positions of the rotating and exit windows on the beamline, the spectroradiometer, and the other UV sources when under test.

A schematic diagram of the laboratory setup is shown in Fig. 1. SURF was equipped with an ultra high vacuum (UHV) chamber section which contained a rotating fused silica window, a limiting aperture, and a fused quartz exit window between the UHV of the beamline and the air atmosphere of the laboratory. Using only a single, fixed exit window would lead to rapid and nonuniform darkening of the window, which is believed to be due to radiation effects. The rotating fused silica window inside the UHV chamber spreads the exposure over an annular area of the window and has been reported to lead to markedly lower changes in the window transmittance [12]. The spectroradiometer was aligned normal to the SURF beam at a nominal distance of 158 cm from the exit window of SURF. The other UV sources to be compared, FEL, D_2_, and ArArc, were placed on an intermediate mount 50 cm from the entrance aperture of the spectroradiometer. These sources were removed to allow the SURF beam to pass unimpeded to the spectroradiometer. All sources could be interchanged relatively rapidly and observed without movement of the spectroradiometer, thus lowering the possibility of responsivity changes in the instrument and alignment errors.

It is important with measurements from disparate sources to adequately baffle the measurement system in order to minimize reflected and scattered light from contaminating the true irradiance signal. Unbaffled, the field of view of the averaging sphere was close to 75°. To limit the field of view of the spectroradiometer, an intermediate baffle with a circular aperture approximately 15 cm in diameter was positioned 25 cm from the entrance aperture of the spectroradiometers averaging sphere. The spectroradiometer was isolated with black cloth behind the intermediate baffle to reduce scattered light. Black cloth baffles were used to screen out other sources of scattered radiation and the alignment laser and SURF beamline were draped with black cloth during lamp measurements. The resultant scattered light from each source was determined using a cylindrical shutter to shadow the entrance aperture of the spectroradiometer just in front of the intermediate baffle. In addition, during measurement of the quartz-halogen irradiance lamps, a black horizontal baffle was used to prevent the spectroradiometer from viewing reflections off the lamp base and mount.

## 3. Spectroradiometer

The design of the spectroradiometer was chosen to minimize errors in the comparison due to differential source properties. An averaging sphere was chosen as the entrance foreoptic to randomize the differential source polarization and geometric properties. The size of the entrance aperture and distance of the averaging sphere to the ArArc, FEL, and D_2_ sources was chosen to be, as closely as possible, identical to those used in their calibration. A double monochromator was used to maximize the out-of-band rejection of the instrument and a “solar blind” photomultiplier tube (PMT) was used to minimize the sensitivity of the instrument to longer wavelength radiation, particularly from the FEL and fluorescence from the instruments optical components.

The spectroradiometer used for this spectral irradiance comparison had a 0.125 m f/4.7 Fastie-Ebert double monochromator. This monochromator was described previously in the two prior source comparisons at SURF [10, 11] and the instrument was reconfigured for this comparison with a new computer control and data acquisition system, detectors, electronics, and foreoptics. The monochromator was equipped with a 3600 groove per millimeter replica ruled grating blazed at 100 nm, and the entrance and exit slits were 5 mm in height and 0.55 mm in width, which produced a spectral bandpass of approximately 1 nm. Spectral scanning was carried out with a lead screw driven sine drive mechanism using a gear-reduced microstepping motor for a wavelength nominal step resolution of 1.95 × 10^−4^ nm per step.

**Fig. 2:**
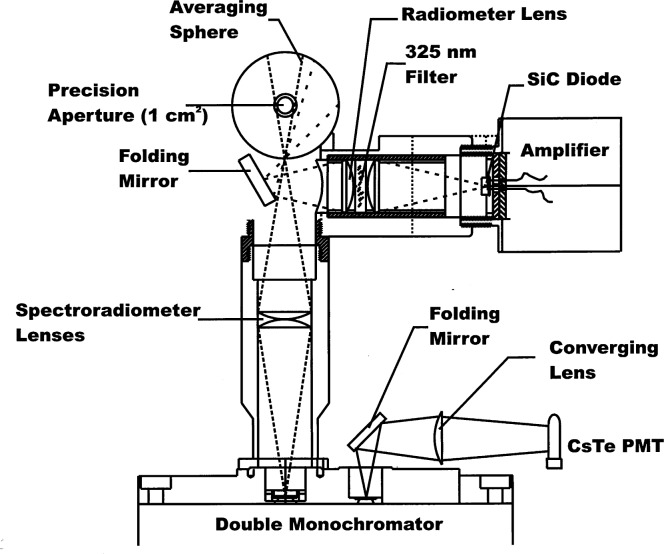
Schematic diagram of the entrance and detector relay optics for the comparison spectroradiometer. The diagram shows the averaging sphere viewing geometry of the double monochromator and the filter radiometer, as well as the relay optics from the monochromator to the CsTe photomultiplier (PMT). The averaging spheres entrance aperture has been rotated 90° for illustrative purposes.

A schematic diagram of the entrance and detector relay optics for the spectroradiometer is shown in Fig. 2. The entrance foreoptic was a 52 mm diameter averaging sphere, with a pressed polytetrafluoroethylene (PTFE) coating. A 1.128 cm diameter precision aperture (circular area of 1.0 cm^2^) was placed at the averaging sphere entrance port and the monochromator viewed the sphere exit port located 908 from the entrance port. An interference filter radiometer viewed the exit port of the averaging sphere just slightly off-axis from the monochromator viewing geometry. This filter radiometer was used primarily to monitor the source stability. The sensor in the filter radiometer was a 3 mm square silicon carbide (SiC) detector. The averaging sphere radiance was imaged onto the SiC detector via a plane folding mirror and two fused silica plano-convex lenses. The interference filter was located between the two lenses and had maximum transmittance of 325 nm with a 20 nm bandpass. The out-of-band transmittance of the interference filter was nominally 1 × 10^−6^ lower than the maximum transmittance of the filter.

The spectroradiometer detector was a “solar blind” CsTe PMT using photon-counting electronics. The deadtime of the photon counting electronics was less than 5 ns with an estimated nonlinearity of less than 0.1 %, particularly at the low counting levels achieved during this experiment. In addition, the data acquisition system contained a 6 1/2 digit multimeter equipped with a ten channel signal multiplexer for measurement of the SURF electron beam current and monitoring other analog signals. The signal from the photodiode in the filter radiometer was converted from current to frequency and the resultant pulses were integrated by the photon counter over the same time interval as the counts from the spectroradiometers PMT. The viewing geometry of the averaging sphere source and the 50 cm source distance from the ArArc, FEL, and D_2_ spectral irradiance standards were nearly identical to those used for the NIST calibrations, thus minimizing the uncertainty due to geometry.

## 4. Source Alignment and Measurement Procedure

The spectroradiometer was aligned with the exiting beam from SURF by retroreflection. The SURF beam was masked with aluminum foil to an approximately 1 mm diameter beam, and the beam was reflected off a glass slide placed over the precision aperture of the spectroradiometer. Final alignment of the entrance aperture normal to the SURF beam was achieved by adjustments of the spectroradiometer mounts until the reflected beam was superimposed on the aperture in the aluminum foil mask. The angular uncertainty in this alignment was on the order of 0.038. In addition, the spectroradiometer was mounted on a computer controlled *X-Y* positioning stage.

This permitted the mapping of the entire SURF beam and precise positioning of the entrance aperture in the center of the SURF beam. This alignment defined the optic axis of the experiment, and the spectroradiometer was not moved afterward. The irradiance of SURF was calculated knowing the distance from the spectroradiometer entrance aperture to the tangent point of the storage ring (1318.5 cm) and the beam current, which was automatically recorded for each SURF measurement.

The NIST scale of spectral irradiance is transferred via the two types of standard lamps: FEL and D_2_. Both lamps are equipped with a medium bipost base and the optical center of the calibrated lamp is located along a centerline between the posts and in the same plane as their front surfaces, and 9.5 cm above their bases.

The position of the spectroradiometer averaging sphere aperture was fixed by retroreflection with the SURF beamline, all alignments of the other UV sources relative to the entrance aperture were accomplished by source positioning. Alignment of the NIST spectral irradiance lamps (FEL, D_2_) relative to the entrance aperture of the spectroradiometer averaging sphere was achieved by using an alignment jig and a HeNe alignment laser [12]. The laser was first aligned normal to and centered on the entrance aperture and then the jig was placed in the lamp mount and aligned parallel to the plane of the entrance aperture. The alignment jig crosshair was centered on the laser beam at the required 50 cm distance from the entrance aperture to the front face of the alignment jig. This alignment positioned the lamps on the optic axis. The distance to the source was estimated to be 50 cm with a 0.05 cm standard uncertainty, resulting in a relative standard uncertainty in irradiance of 0.2 %. The lamps and jig were mounted in specially designed lamp sockets that fixed the lamp height and to which the current and voltage leads of the lamp power supply system were attached.

The current from the power supply to the FEL lamps was controlled by an external voltage supplied by a computer-controlled 16 bit digital-to-analog converter [13]. The current through the lamps was determined by measuring the voltage across a calibrated shunt resistor with a 6 1/2 digit multimeter, and the current from the power supply was monitored and adjusted by a computer to maintain the desired value. The lamp power supply system was operated by a separate computer than that used to control the spectroradiometer. The standard uncertainty of the lamp current was less than 0.05 mA. The voltage across the lamp filament was also monitored.

The electrical power supply system used to operate the D_2_ lamps for this experiment was the same as used in their calibration in FASCAL [3]. It consisted of a heater filament dc power supply (10 V, 1.2 A) and a main dc power supply (500 V, 0.3 A). To start the lamp the filament was heated for 30 s at 10 V. The 500 V was then applied, striking the arc. The heater voltage was then reduced to 7 V for operation of the lamp. The lamp current and voltage were monitored and adjusted over the course of the experiment.

The HeNe alignment laser was also used to align the axis of the argon miniarc to the optic axis of the spectroradiometer. Alignment procedures were similar to that employed with the FEL and D_2_ lamps. The distance from the spectroradiometers entrance aperture to the center of the arc position was 50 cm with a standard uncertainty of 0.1 cm, resulting in a relative standard uncertainty in irradiance of 0.4 %. The current through the argon miniarc was determined by measuring the voltage across a calibrated shunt resistor with a voltmeter, and the current from the power supply was adjusted manually to maintain the desired value (40.0 A) with a standard uncertainty of 0.02 A. Scientists from the APD supplied the ArArc, the necessary equipment for its operation and assisted in the alignment and operation of the source. The argon miniarc was operated without a window, and was measured on two succeeding days over the course of the experiment; two signal scans and a scattered light scan were carried out on each day.

Spectral scans of UV sources were from 200 nm to 305 nm at 5 nm intervals. The photon counter integration time was 10 s with five repeat signal measurements at each wavelength. The total scan duration was approximately 20 min. For sources which required a warm-up time (FEL, D_2_, ArArc), a scattered light scan was taken initially with the cylindrical shutter while the source was thermally equilibrating, followed by a single signal scan. For observations of the irradiance from SURF, three signal scans and one scattered light scan were taken. The dark counting rate of the PMT was typically less than one count per second and scattered light measurements from the shuttered sources were on the order of 1 to 3 counts per second. Scans of UV line sources, Hg and Zn emission lamps, were carried out every day of the comparison and no statistically significant change in the wavelength calibration of the UV spectroradiometer was observed. The standard uncertainty of the wavelength of the spectroradiometer calibration was less than 0.04 nm over the entire wavelength range. The maximum relative standard uncertainty of the responsivity due to uncertainty in wavelength uncertainty was 0.5 %, 0.2 % and 0.4 % at 210 nm for the ArArc, D_2_ and SURF, respectively, and 0.2 % at 250 nm for the FEL.

Calculation of SURF irradiance at the spectroradiometer position necessitated the measurements of the transmittance of two optical elements: the rotating fused silica disk and the fused silica chamber exit window. These elements were paired and referred to as a window set. At the end of a days measurements the disk and exit window were removed and their transmittance measured on a separate instrument. Two sets of windows were used for the experiment in order to interchange the windows and evacuate the chamber before experiments began the next morning.

## 5. Results

**Fig. 3:**
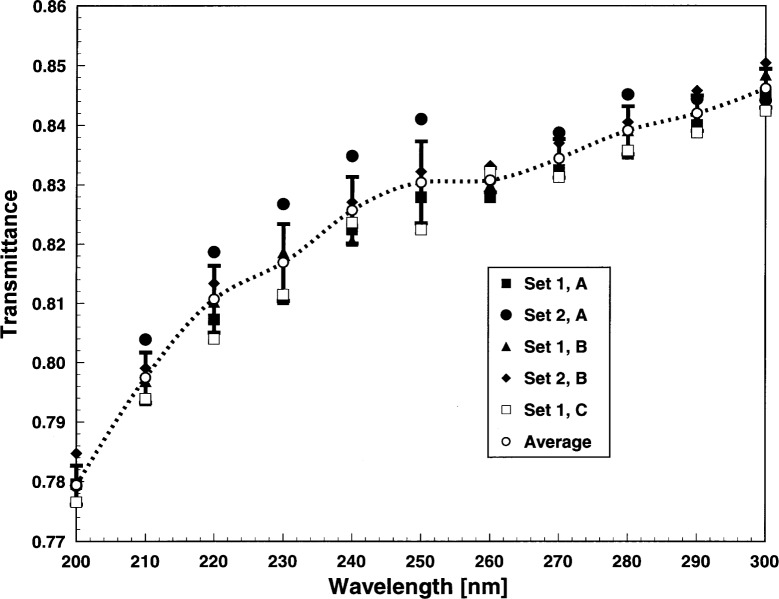
Transmittance as a function of wavelength of the SURF window sets used in the comparison experiment. There were two Window Sets (1,2), with a window set consisting of a rotating fused silica disk and the exit window. Window Set 1 was measured three times over the course of the experiment and Window Set 2 was measured twice. The average of all window set transmittance measurements is shown and the vertical bars are the standard deviation. The dotted line is a cubic spline fit for illustrative purposes.

The measured spectral transmittances of the two window sets over the course of the experiment are shown in Fig. 3. The transmittance of window set 1 was measured three times (A, B, C) and window set 2 was measured twice (A, B). There was no statistically significant change in the transmittance of window set 1 over the course of the experiment and the variation in measured transmittance appears to be due to random variations in the transmittance measurement. Window set 2 appeared to change in transmittance, but this observed change could have been the result of problems in the first transmittance measurement of window set 2. The average and standard deviation (error bars) of all window set transmittances are also shown in Fig. 3. Due to the small number of transmittance measurements, it was decided to use the average of the five window set transmittances in the calculation of the SURF irradiance for the comparison experiment.

**Fig. 4:**
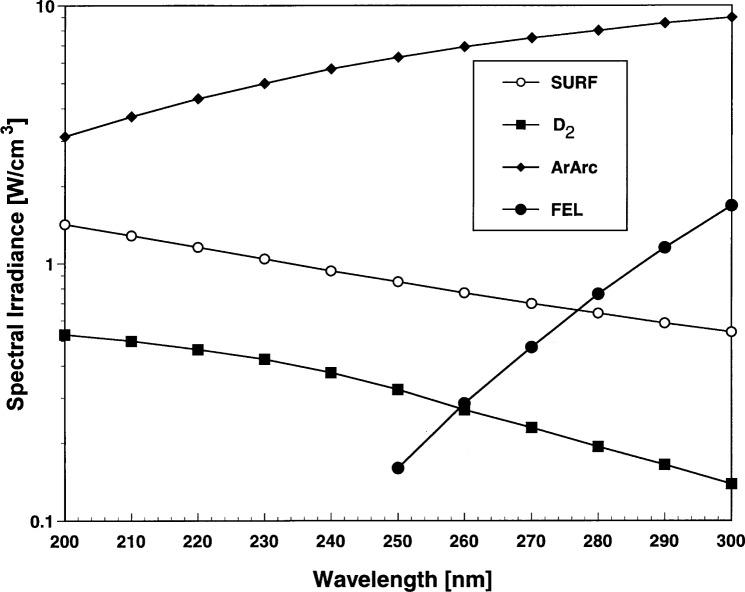
Nominal spectral irradiances as a function of wavelength from the different types of ultraviolet sources evaluated in the comparison.

Typical spectral irradiances for each type of source used in the experiment are shown for comparison purposes in Fig. 4. The values for SURF were calculated for a beam current of 100 mA and the average window transmittance. The spectral irradiance values for ArArc, FEL, and D_2_ were the calibrated values for sources observed during the comparison. The relative spectral shapes of the D_2_ and SURF irradiance spectra are similar, but differ markedly from the ArArc and the FEL. The irradiance from the ArArc is greater than SURF and the shape approximates a 10 000 K blackbody spectrum in this spectral region. The irradiance from the FEL is less than SURF and the spectral shape approximates a 3000 K blackbody.

**Fig. 5:**
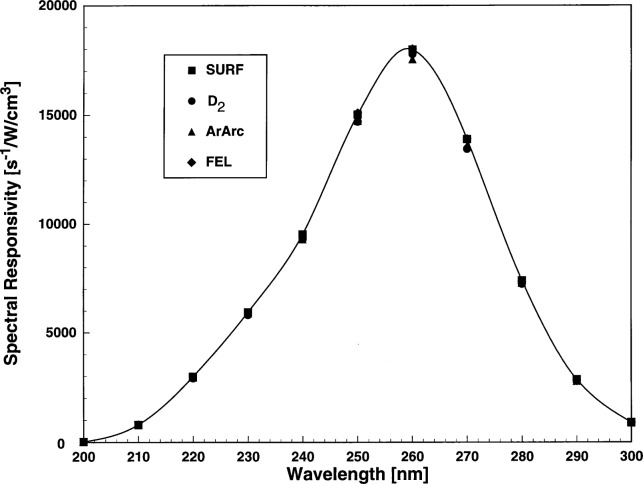
Spectral responsivity of the spectroradiometer as a function of wavelength as determined with ultraviolet sources evaluated in the comparison (SURF, FEL, ArArc, D_2_). Spectral responsivity uncertainties are smaller than the symbols used for the individual values.

The irradiance values for the ArArc, FEL, and D_2_ were supplied by the NIST calibration services in the OTD and APD. The SURF spectral irradiance is calculated from the instantaneous beam current, the distance of the entrance aperture of the spectroradiometer to the storage ring tangent point and the transmittances of the windows. For each source, the spectral responsivity of the spectroradiometer was calculated from the net signal and the source spectral irradiance. The net signal was the signal scan minus the scattered light scan. In the case of SURF, three scans of the signal were taken and one scattered light scan on each day of the comparison. The instrument spectral responsivity was determined for each SURF scan and the average spectral responsivity for that day was determined. The spectral responsivity of the spectroradiometer as determined for each irradiance source is shown in Fig. 5. The solid line is a spline fit to the data set for purposes of illustration. The decreased responsivity for wavelengths longer than 260 nm is largely the result of the response of the CeTe PMT. The response at wavelengths shorter than 260 nm is due to the wavelength dependent changes of the focal properties of the fused silica lens in the input optics of the spectroradiometer. The placement of the lenses was optimized for 270 nm.

For this experiment short term stability of the spectroradiometer was assumed for all scans taken from different sources over a single day. Small variations of less than 2 % in the day to day instrument responsivity from all sources were observed. These changes were also detected in the corresponding responsivity of the filter radiometer, indicating that small changes were probably occurring in the reflectivity of the averaging sphere. To scale the instrument spectral responsivity values which changed by a factor of approximately 23 from 210 nm to 300 nm and to compensate for small fluctuations in the daily absolute responsivity of the instrument, the relative responsivity was calculated by ratioing all source responsivities by the responsivity from SURF on that day. SURF was chosen as the normalization factor for several reasons: 1) SURF is a primary standard while the lamps and arc sources are secondary standards; 2) the spectral irradiance from SURF covered the entire wavelength range; and 3) multiple observations of SURF were carried out on each day of the comparison. The problem in the choice of SURF as the reference is, due to the uncertainty in the determination of the window set transmittance, unknown wavelength-dependent effects will introduce variation in the final results.

A comparison between the NIST spectral irradiance scales can be obtained by determining the ratio of the instrument responsivity with SURF irradiances to the instrument responsivity using the other irradiances from other sources [15]. The simplified measurement equation for the signal using SURF is

$$S_{\text{SURF}}(\lambda )=E_{\text{SURF}}(\lambda )\;R_{\text{SURF}}(\lambda ),$$ (5.1)

where λ is the wavelength, *S*_SURF_(λ) is the output signal of the photon counting circuit, *E*_SURF_(λ) is the spectral irradiance of the source, and *R*_SURF_(λ) is the spectral irradiance responsivity function of the instrument. Likewise, for another irradiance source, indicated by the subscript SOURCE,

$$S_{\text{SOURCE}}(\lambda )=E_{\text{SOURCE}}(\lambda )\;R_{\text{SOURCE}}(\lambda ),$$ (5.2)

In Eq. (5.2), *E*_SOURCE_(λ) is the spectral irradiance assigned to the source by its calibration. Assuming the actual responsivity to be *R*_SURF_(λ), Eq. (5.2) can be rewritten as

$$S_{\text{SOURCE}}(\lambda )=E\prime_{\text{SOURCE}}(\lambda )\;R_{\text{SURF}}(\lambda ),$$ (5.3)

where *E*′_SOURCE_(λ) is the irradiance of the other source based upon the SURF determined responsivity. Equating Eqs. (5.2) and (5.3) and rearranging yields

$$\frac{R_{\text{SURF}}(\lambda )}{R_{\text{SOURCE}}(\lambda )}=\frac{E_{\text{SOURCE}}(\lambda )}{E\prime_{\text{SOURCE}}(\lambda )}$$ (5.4)

Therefore, the ratio of the calibrated spectral irradiance of a source to the spectral irradiance of that source based on SURF can be determined by taking the inverse of the experimentally determined responsivity ratio.

**Fig. 6:**
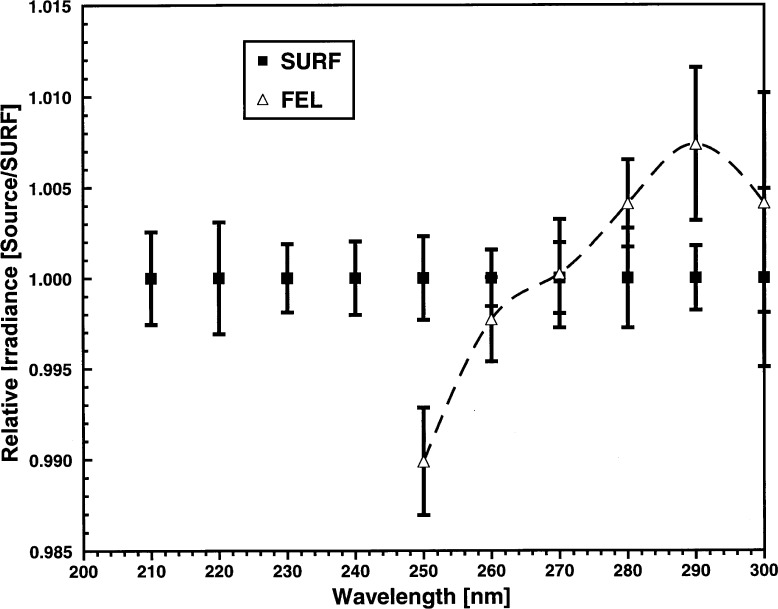
Relative irradiance ratio as a function of wavelength for the UV sources SURF and the FEL lamps. The measurements were carried out over 4 successive days and comprised 12 measurements from SURF and six measurements from two FEL lamps. The cumulative results for the 4 days were averaged and the standard deviation determined. The symbols are the averages and the error bars are the standard deviations. The dashed line is a cubic spline fit to the FEL ratios for illustrative purposes.

The data for this irradiance comparison were taken over the course of 4 days, October 24–27, 1995. The data for all observations of the SURF and FEL sources are shown in Fig. 6. There were 12 SURF spectral scans over the course of the four days, the means and standard deviations of the irradiance ratios are shown as the symbols and vertical bars. Each ratio was determined by taking the individual determinations of the spectroradiometers spectral responsivity and dividing by the average spectral responsivity as determined by SURF on that day. From Eq. (5.4), the relative irradiance ratio is the inverse of the responsivity ratio. By definition, the average SURF/SURF irradiance ratio was 1 for the SURF measurements and it was reproducible to within 0.3 %. The higher standard deviation at 300 nm was due to the much lower signal from SURF and the much lower instrument responsivity at that wavelength.

There were six spectral scans from two FEL type lamps (F-305, F-315) over the course of the 4 day experiment. The means and standard deviations of the FEL results are shown by the symbols and vertical bars in Fig. 6. The dashed line is a spline fit through the FEL/SURF data for the purposes of illustration. The reproducibility of the FEL/SURF ratio is approximately the same as for the SURF results, but with a small wavelength dependence.

**Fig. 7:**
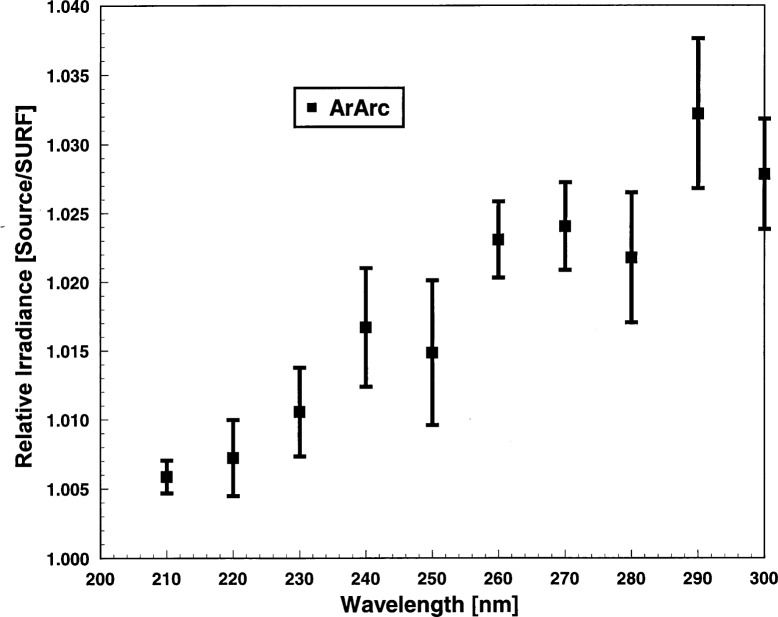
Relative irradiance ratio as a function of wavelength for the ArArc. The measurements were carried out over 2 successive days comprising four spectral scans of an argon miniarc. The filled square symbols are the averages and the vertical bars are the standard deviations.

The observations of the ArArc for the irradiance comparison occurred on 2 days, October 24 and 25, 1995. On each day there were two spectral scans of the ArArc. The resultant four relative responsivities were averaged and the results are presented in Fig. 7. The means and standard deviations of the ArArc results are shown by the symbols and vertical bars in Fig. 7. The ArArc results are reproducible to within 0.5 %, but they are consistently higher than expected value of unity at all wavelengths. This indicates a possible bias in the assignment of ArArc irradiance. The discrepancy with SURF is greatest at the longer wavelengths; this trend was also observed with the FEL lamps (Fig. 6).

**Fig. 8:**
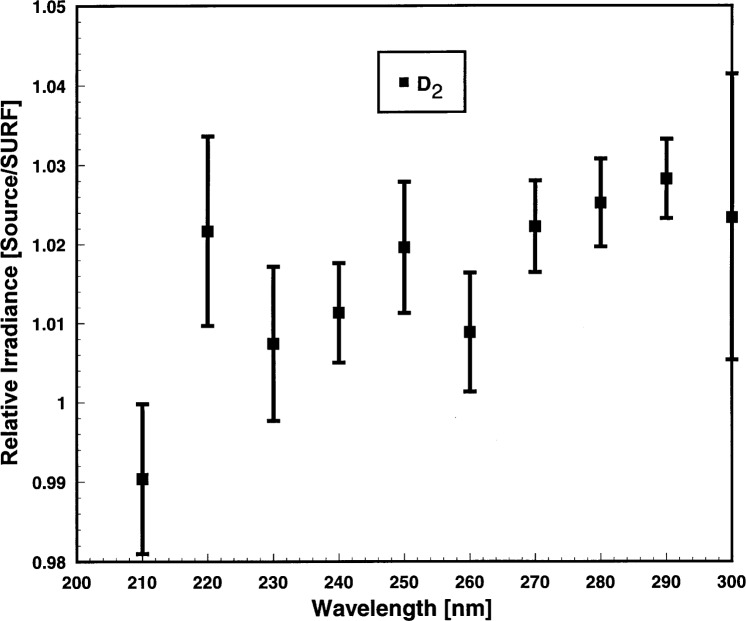
Relative irradiance ratio as a function of wavelength for the D_2_ lamps. Measurements were carried out over 4 successive days comprising six spectral scans from three deuterium lamps. The filled square symbols are the averages and the vertical bars are the standard deviations.

The observations of the D_2_ lamps occurred on all 4 days of the comparison. There were six spectral scans of three D_2_ lamps, one of which is a NIST working standard. The resultant six relative irradiances were averaged and the results are presented in Fig. 8. The means and standard deviations of the D_2_/SURF ratio are shown by the symbols and vertical bars in Fig. 8. The D_2_ results were reproducible to between 0.5 % to 1.2 %. A similar wavelength-dependent trend was observed in the D_2_ irradiance ratios as was observed in the FEL (Fig. 6) and in the ArArc ratios (Fig. 7).

Table 1 lists the spectral irradiance relative standard uncertainties, in percent, from the four sources SURF, FEL, ArArc, and D_2_ and from the spectroradiometer at 250 nm. The NIST assigned spectral irradiance relative standard uncertainties for each source are the calibration values. The uncertainties due to random variations of the UV sources are represented by the day-to-day reproducibility of the irradiance ratios from Figs. 6, 7, and 8. The reproducibility in Table 1 is that expected upon relighting of the source. On each day of the comparison, the irradiances of the ArArc and D_2_ were not adjusted to the FEL or SURF irradiances, the aim being to compare the assigned irradiances independent of other irradiance standards. The total uncertainties are the root sum of squares of the component uncertainties.

Table 2 summarizes the observed irradiance differences and combined relative standard uncertainties, in percent, at 250 nm for each UV source pair. The irradiance differences were calculated from the appropriate source pair irradiance ratios minus one. The combined standard uncertainties in Table 2 are calculated for each source pair as the quadrature sum of the total values from Table 1. The observed irradiance differences at 250 nm from Table 2 are within the respective combined relative standard uncertainties for all source pairs. Furthermore, the observed irradiance differences at all comparison wavelengths and source pairs were within the relative standard uncertainties.

## 6. Conclusions

The NIST spectral irradiance sources in the ultraviolet region from 210 nm to 300 nm are internally consistent and agree within their combined standard uncertainties. These results agree with the previous results of Kostkowski and colleagues [10, 11]. The results disagree with the recent results from the CCPR air UV intercomparison [8] and with the detector-based UV source comparisons of Foukal and Jauniskis [15, 16]. Foukal and Jauniskis measured NIST UV sources with a filter radiometer calibrated with a standard uncertainty of 0.23 % using a 257 nm laser and a cryogenic electrical substitution radiometer. These detector-based irradiance assignments disagreed with NIST by approximately 9 % from SURF, 2 % with FEL lamps, and 7 % from D_2_ lamps. These disagreements by Foukal and Jauniskis with both the NIST assigned values and the results of the current work probably trace to inadequate radiometer straylight rejection, source geometrical factors, SURF window degradation, and source instability.

The design of the transfer spectroradiometer appeared to minimize the biases that can arise from known differences in sources, such as geometry, spectral distributions, stray light and polarization. The experimental experience with rotating fused silica disk was not sufficient for a assessment of whether the degradation of the SURF exit window was decreased. Improved stability of the exit window could result in reduced uncertainties in the use of SURF as an in-air calibration source, as the variable temperature blackbody is currently used in FASCAL. The results indicate that a wavelength dependent bias exists; this trend was seen for all three source ratios. Future experiments should be designed to isolate the origin of the observed wavelength dependent trends. Possible explanations include the window transmittance measurement, the transfer spectroradiometer, and the spectral irradiance scales themselves. Future experiments should also incorporate artifacts directly traceable to absolute detector scales at NIST.

## Figures and Tables

**Table 1:** Experimental relative standard uncertainties, in percent, associated with the uncertainty from the sources (SURF, FEL, ArArc, and D_2_) and from the spectroradiometer at 250 nm. Total is the relative standard uncertainty in spectral irradiance for each source and the spectroradiometer, and is the quadrature sum of the component uncertainties

Component	Relative standard uncertainty (%)				

	Source				Spectroradiometer
	SURF	FEL	ArArc	D_2_	
Experimental					
Random contributions	0.2	0.3	0.5	0.8	
Systematic contributions					
Alignment	0.1	0.08	0.5	0.08	
Distance	0.05	0.2	0.4	0.2	
Window transmittance	0.83				
Wavelength					0.2
Linearity					0.1
Receiver geometry					0.1
Fluorescence					0.1
Irradiance assignment					
Calibration	0.5	0.91	3.0	1.6	
Reproducibility		0.05	1.0	2.0	

Total	1.00	0.98	3.26	2.69	0.26

**Table 2:** Summary of the UV source pair irradiance differences and combined relative standard uncertainties, in percent, at 250 nm. The combined relative standard uncertainties for each source pair is the quadrature sum of the total source and spectroradiometer uncertainties from Table 1

Source pair	Observed irradiance difference (%) (SURF-Source)	Combined relative standard uncertainty (%)
SURF and FEL	−1.1	1.42
SURF and ArArc	1.48	3.42
SURF and D_2_	1.96	2.88
